# Supplementary material for: A Predator on the Doorstep: Kill Site Selection by a Lone Wolf in a Peri-Urban Park in a Mediterranean Area
Source: Animals (Basel). 2023 Jan 30;13(3):480. doi: 10.3390/ani13030480 (PMC9913258; doi:10.3390/ani13030480)
Supplement: Supplementary file 1 [file animals-13-00480-s001.zip › FigureS1-Zoom_on_open_areas_present_in_the_SanRossore_Estate.pdf]

Supplementary Figure S1.
Zoom on open areas in San Rossore Estate

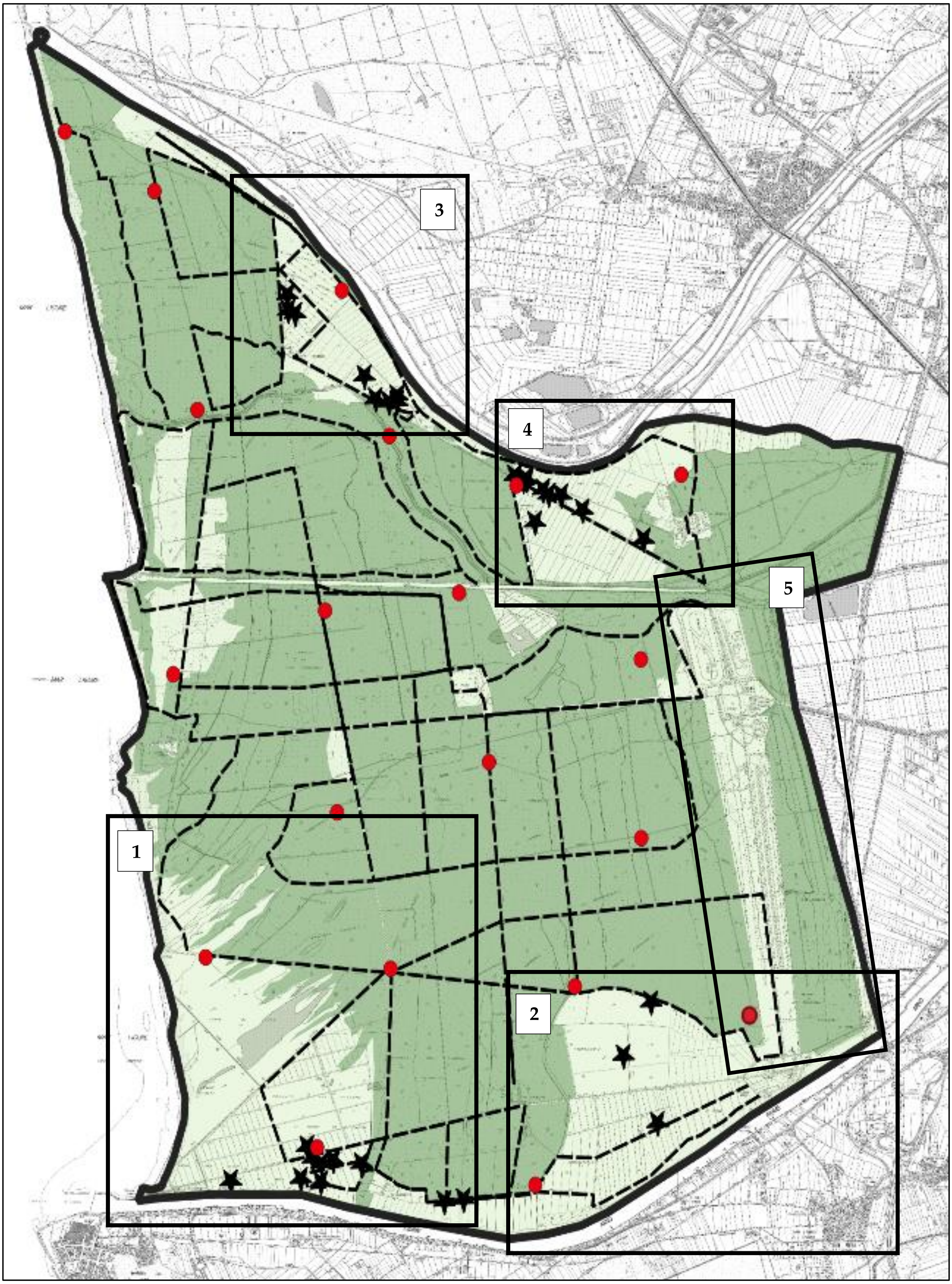

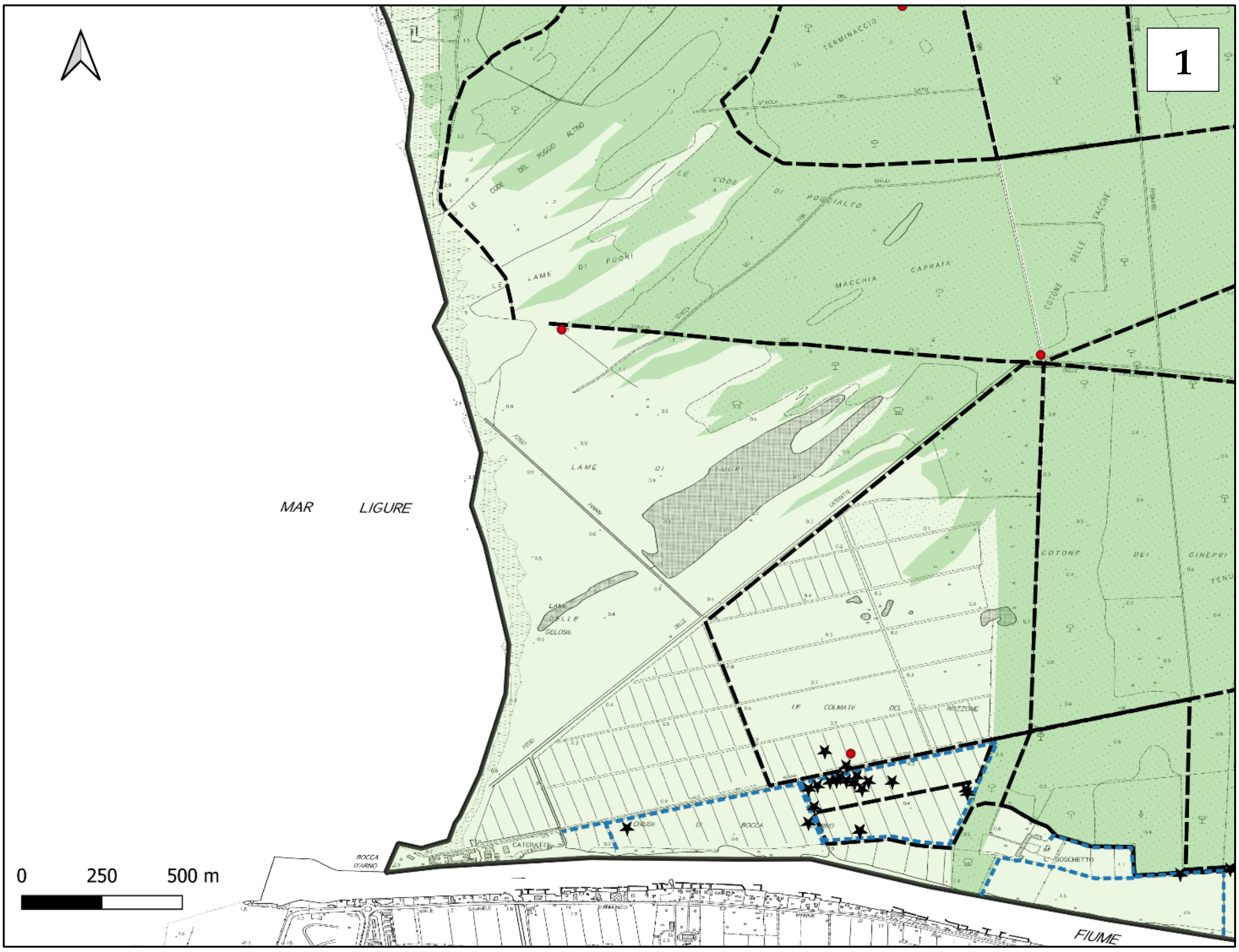
1
MAR LIGURE
LAME DI FUORI
LE COLMATE DEL ROZZONE
FIUME
0 250 500 m

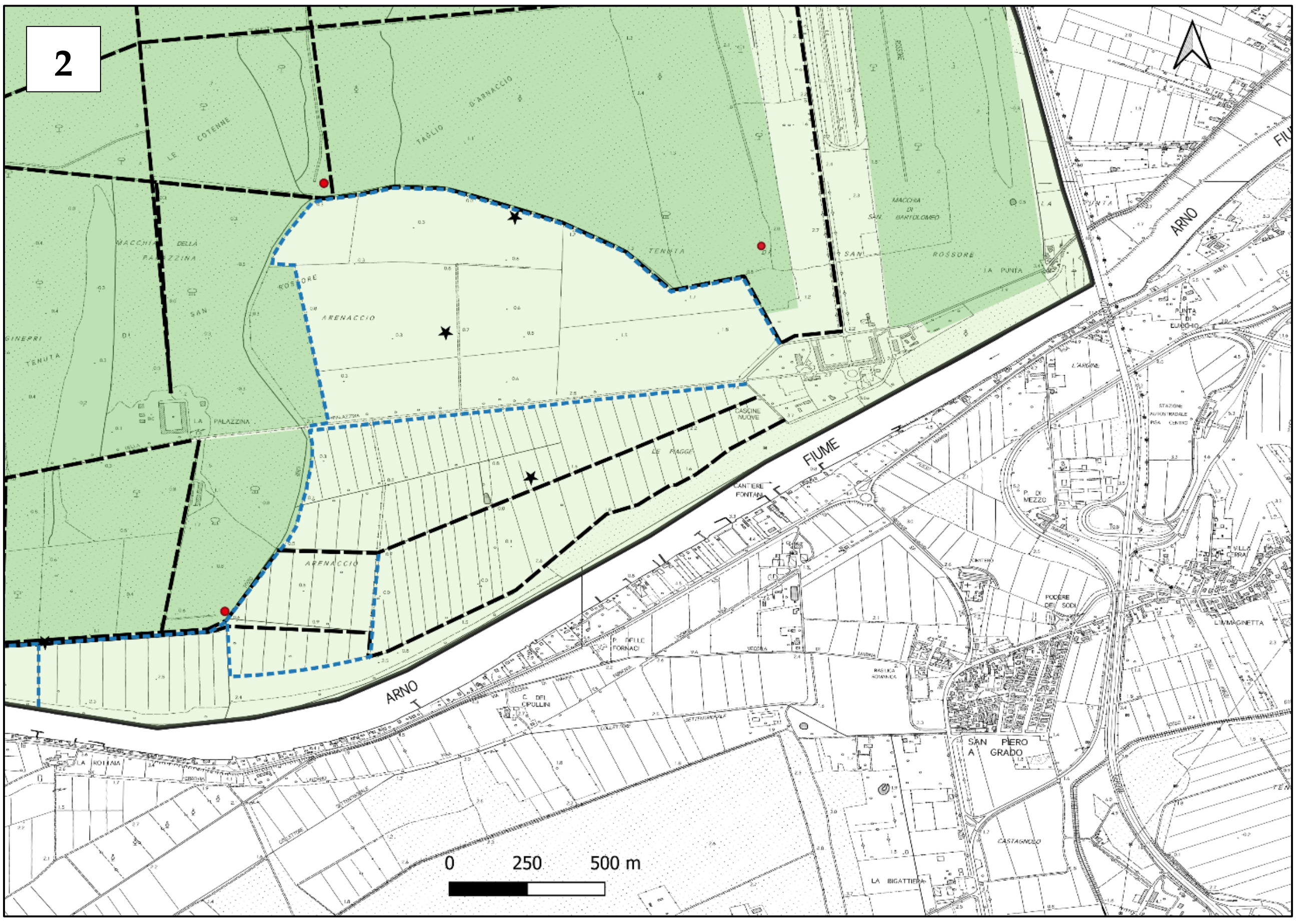
2
ARENACCIO
FIUME
ARNO
SAN PIERO A GRADO
0 250 500 m

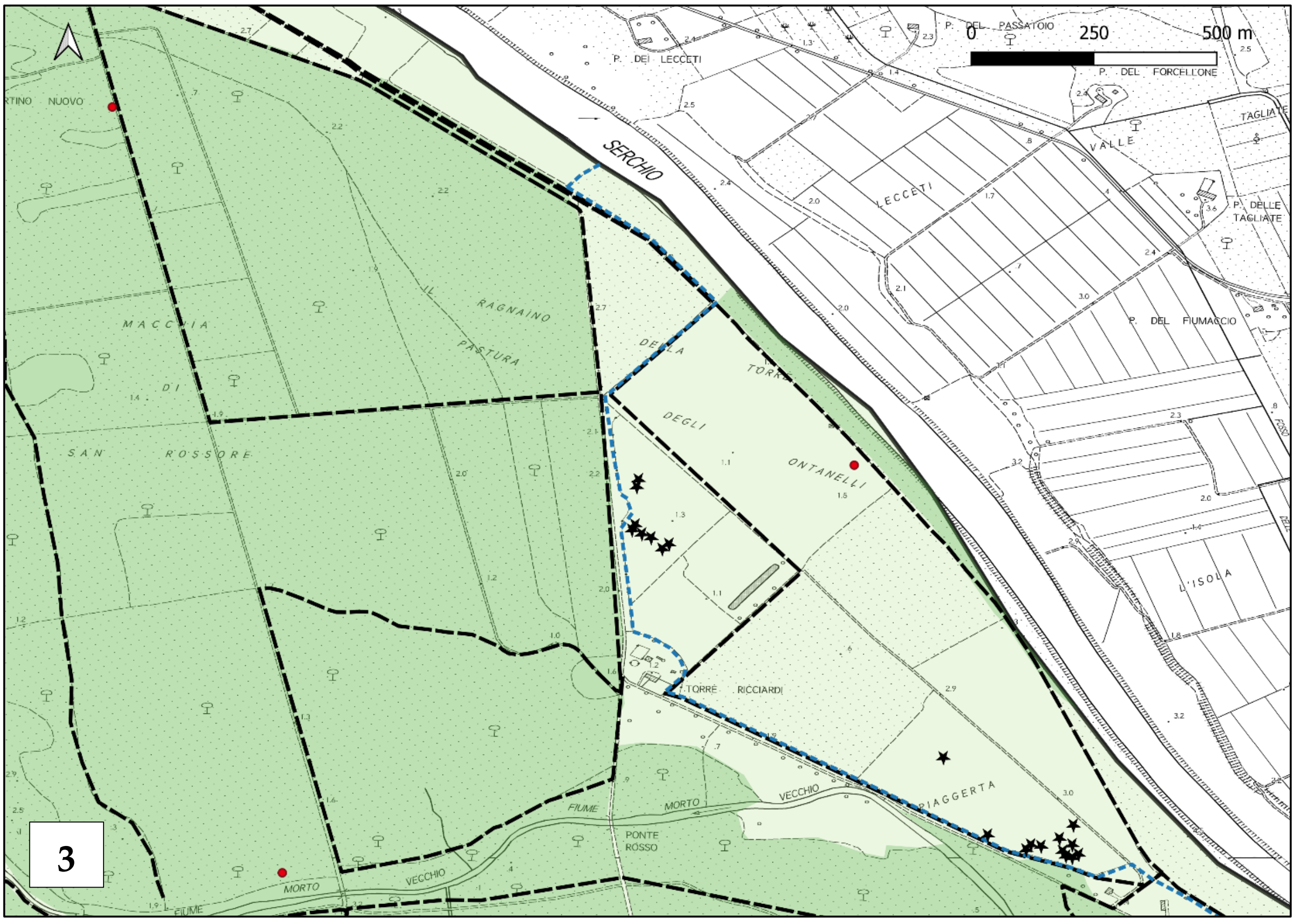
3
0 250 500 m
SERCHIO
P. DEI LECCETI
P. DEL PASSATOIO
P. DEL FORCELLONE
VALLE
TAGLIATE
P. DELLE TAGLIATE
LECCETI
P. DEL FIUMACCIO
L'ISOLA
MACCHIA DI SAN ROSSORE
IL RAGNAINO
PASTURA
DELLA TORRE DEGLI ONTANELLI
TORRE RICCIARDI
PIAGGERTA
FIUME MORTO VECCHIO
PONTE ROSSO
MORTO VECCHIO

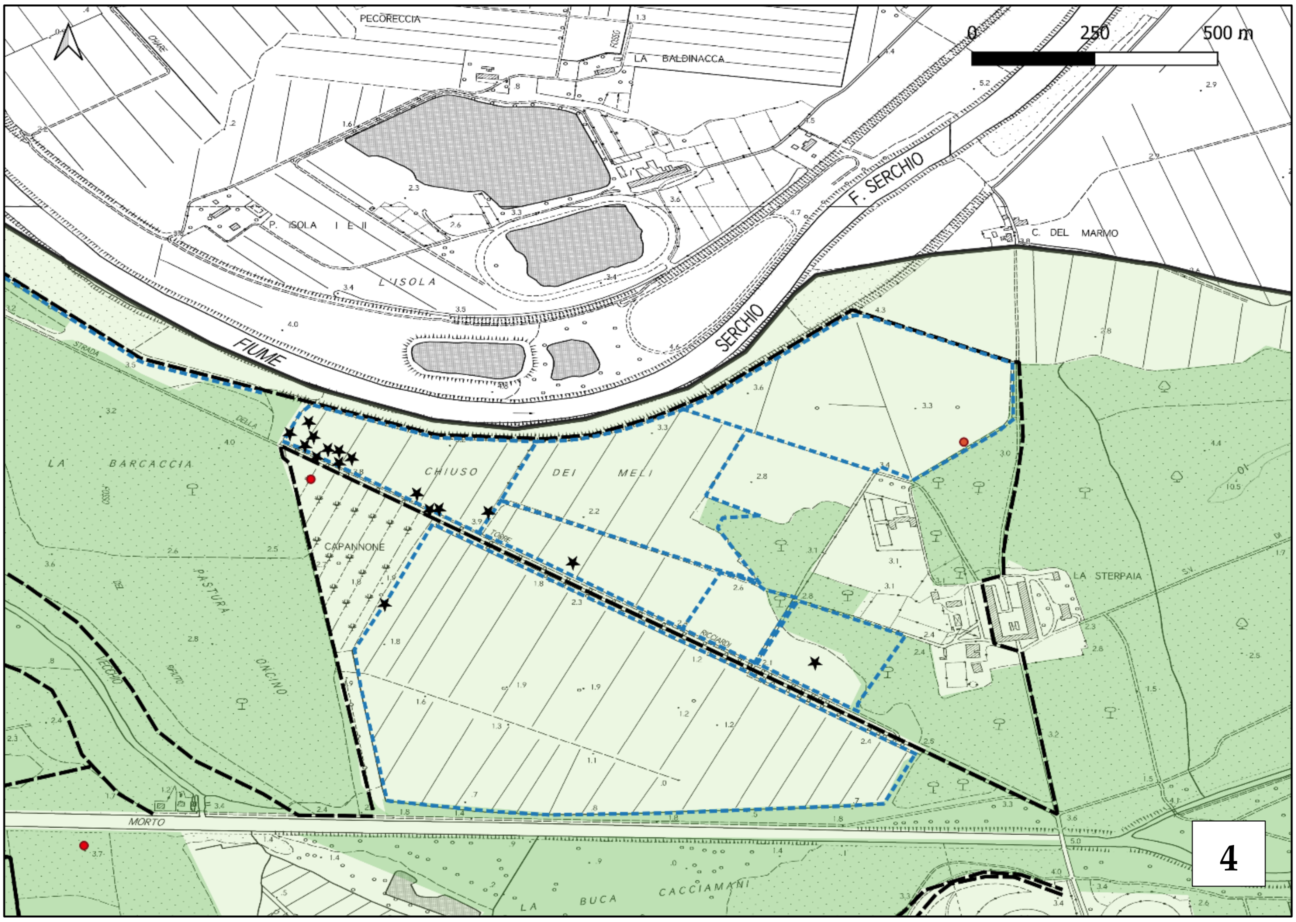
4
0 250 500 m
PECORECCIA
LA BALDINACCA
P. ISOLA I E II
L'ISOLA
FIUME SERCHIO
F. SERCHIO
C. DEL MARMO
LA BARCACCIA
CHIUSO DEI MELI
CAPANNONE
PASTURA OLCINO
LA STERPAIA
MORTO
LA BUCA CACCIAMANI

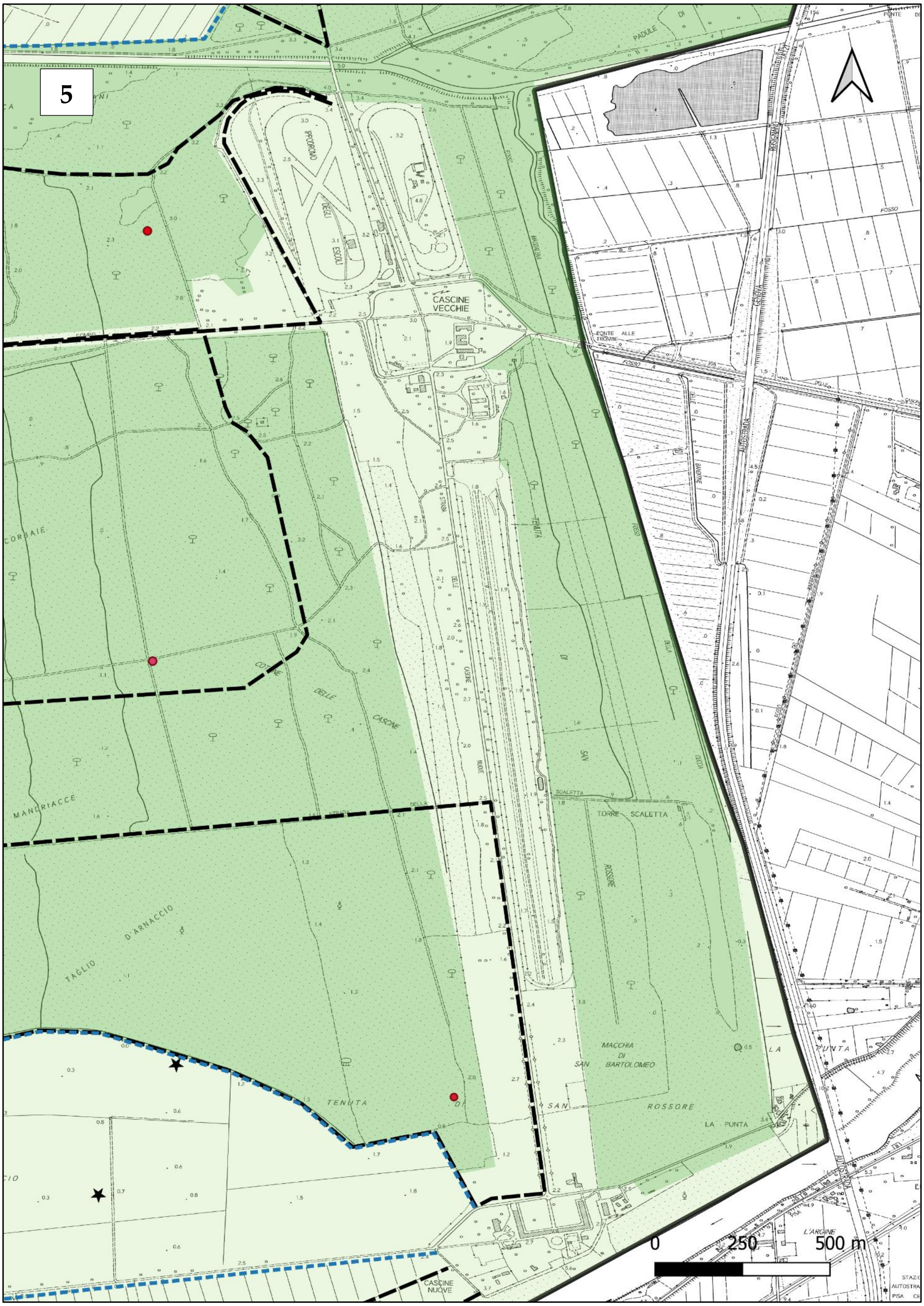
5
CASCINE VECCHIE
IPPODROMO DEGLI ESCOLI
PADULE
PONTE ALLE TROMBE
FOSSO
CORBAIE
MANDRIACCE
TAGLIO D'ARNACCIO
COTONE DELLE CASCINE
STRADA DELLE CASCINE NUOVE
TENUTA DI SAN ROSSORE
TORRE SCALETTA
MACCHIA DI SAN BARTOLOMEO
LA PUNTA
CASCINE NUOVE
L'ARGINE
AUTOSTRADA
0
250
500 m

Figure S1- Open areas present in the San Rossore Estate**.** In the five boxes (1-5) fences are shown (blue dashed lines), together with transects (black dashed lines) camera traps (red dots) and kills (black stars). All predations were found in open areas, but most of them near the fences; only a very few preyed deer were detected in the remaining part of open areas.
