# Supplementary material for: A Predator on the Doorstep: Kill Site Selection by a Lone Wolf in a Peri-Urban Park in a Mediterranean Area
Source: Animals (Basel). 2023 Jan 30;13(3):480. doi: 10.3390/ani13030480 (PMC9913258; doi:10.3390/ani13030480)
Supplement: Supplementary file 1 [file animals-13-00480-s001.zip › FigureS2_Morphological_traits_used_for_wolf_identifcation.pdf]

Supplementary Figure S2

Morphological traits used for wolf identification

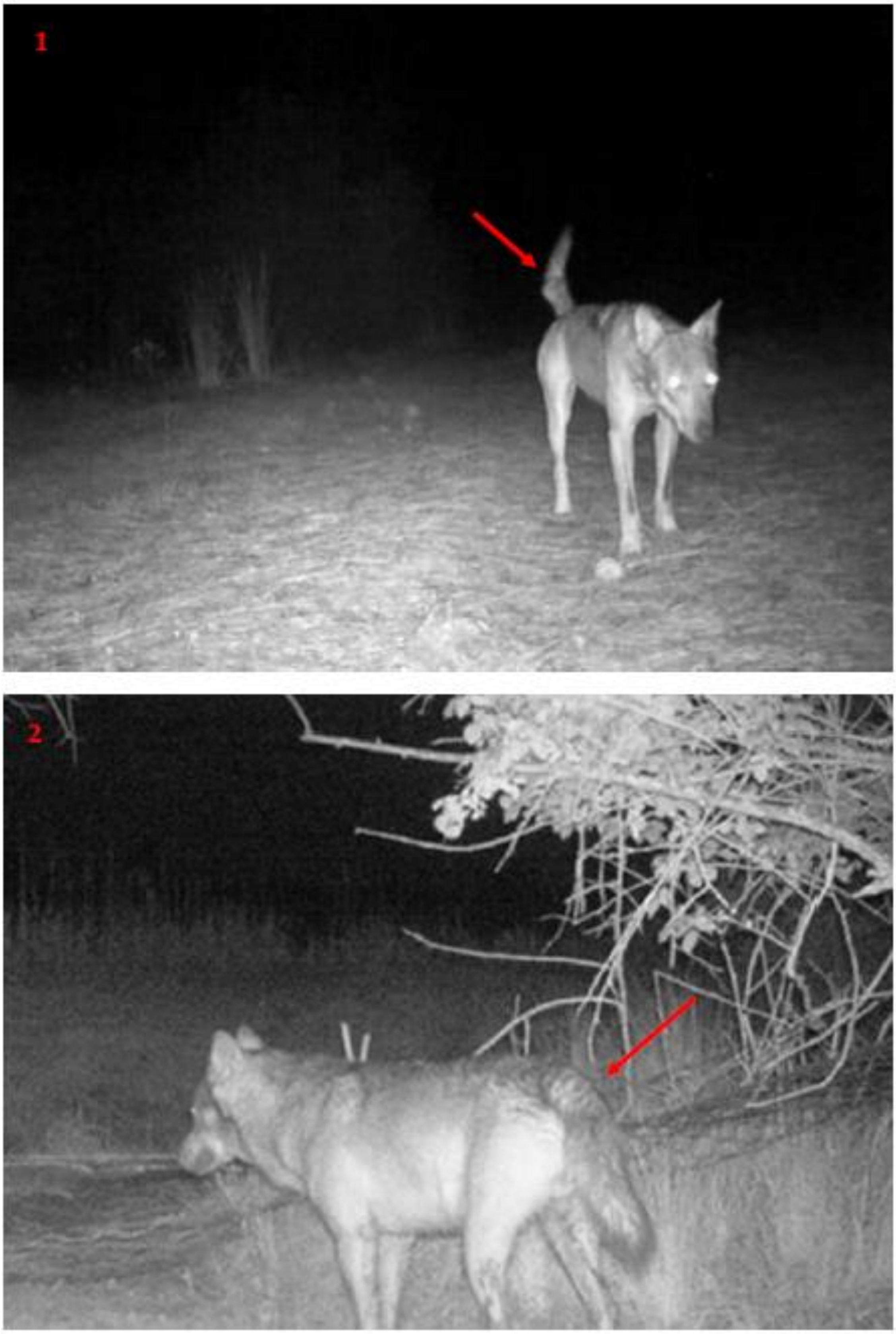

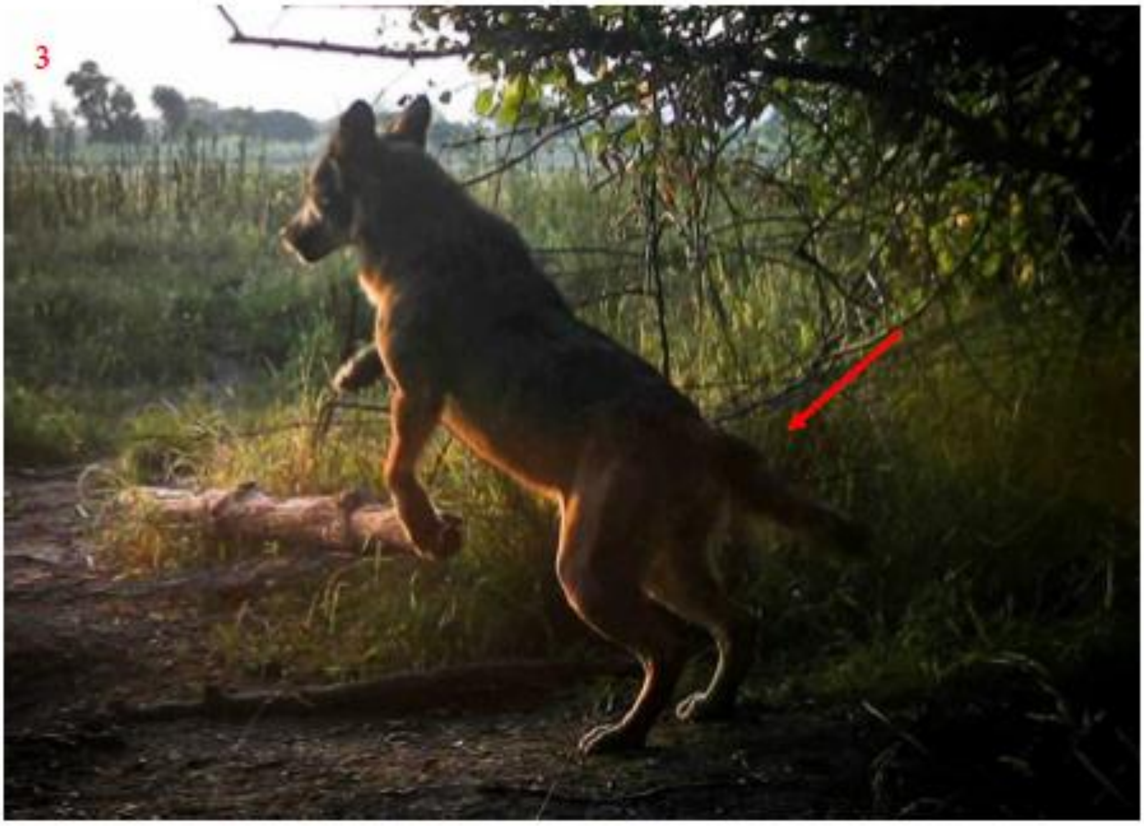
3

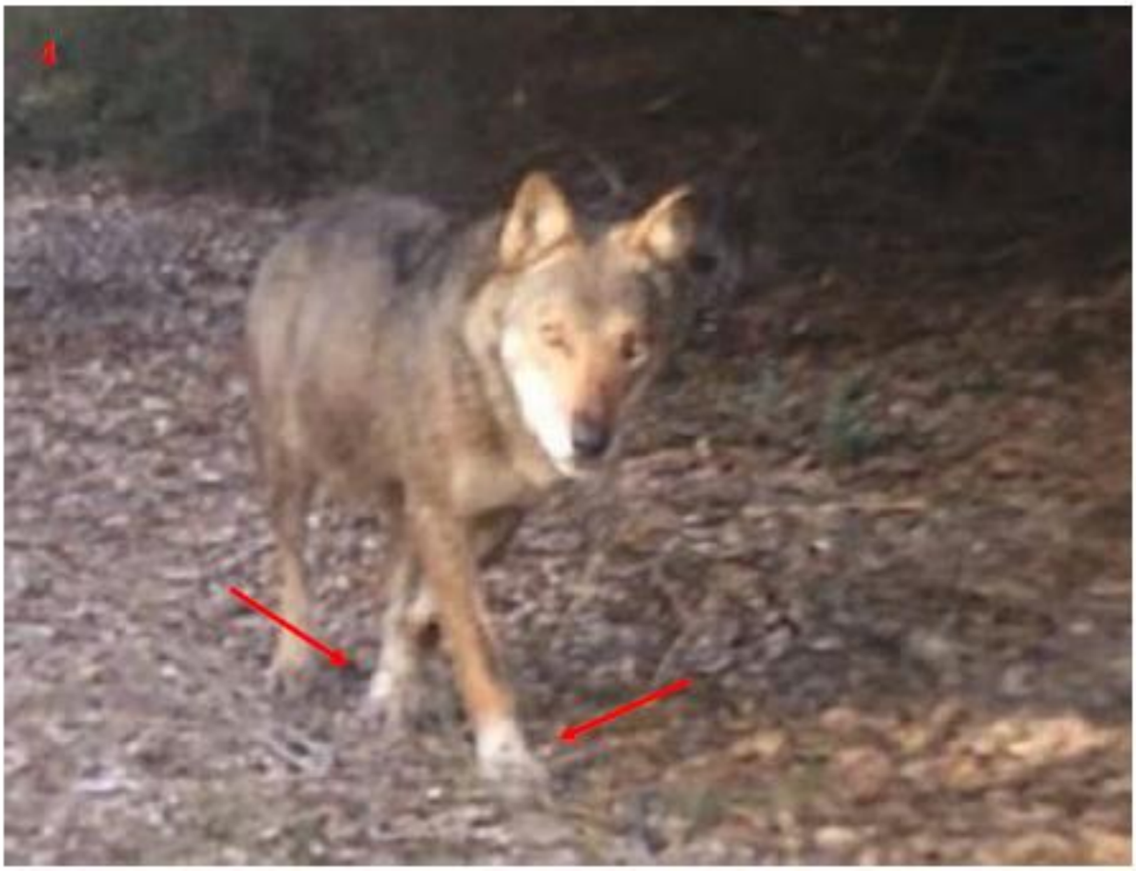
4

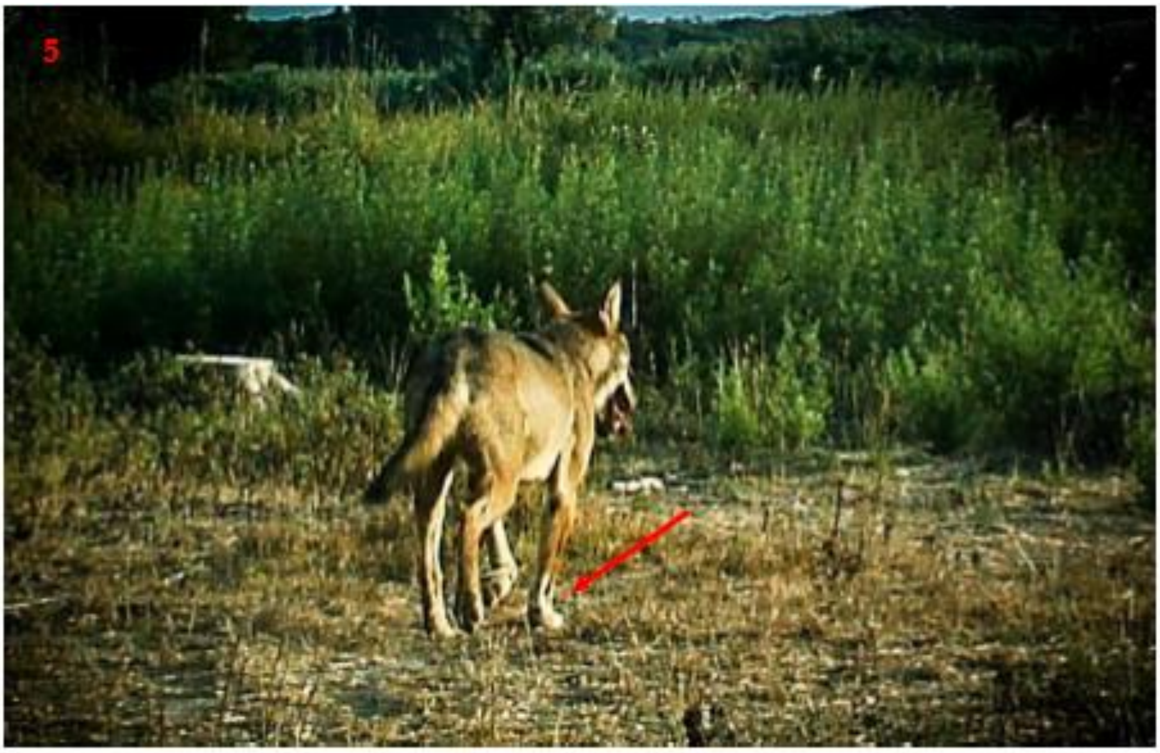

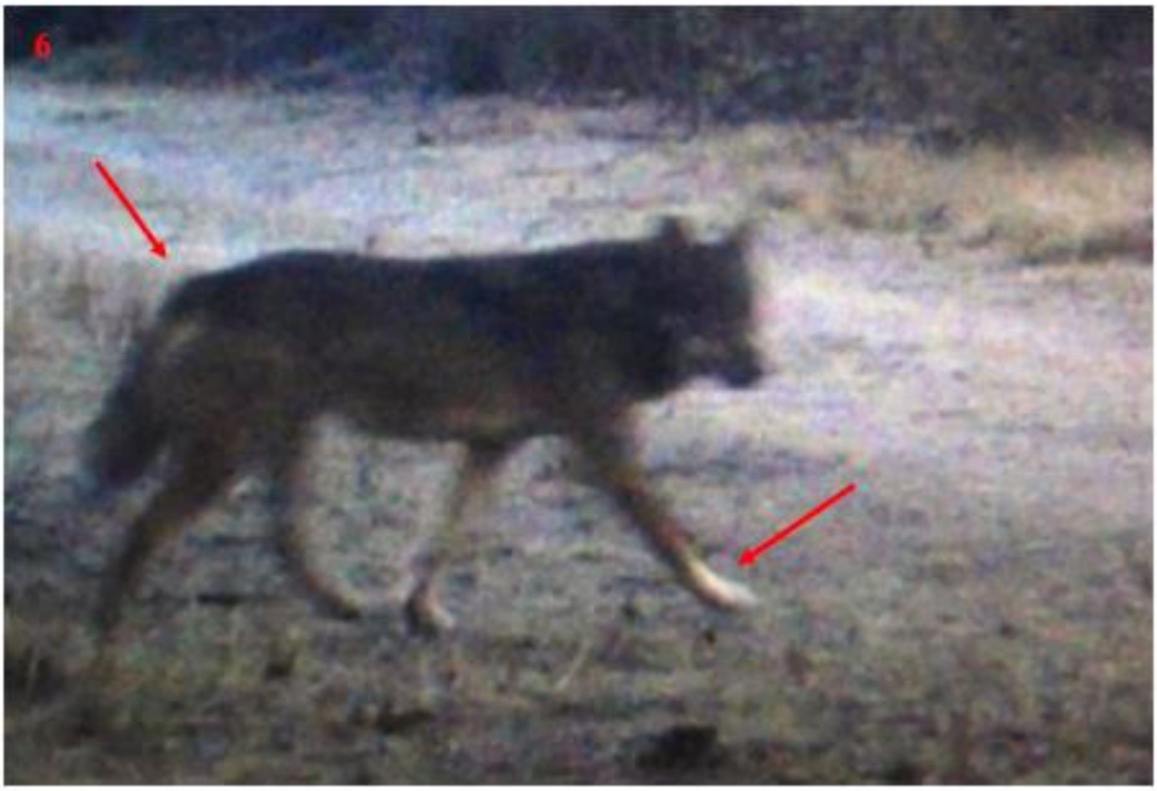

Figure S2: In the pictures number 1, 2, and 3 the red arrow indicate the short tail with an abnormal curve at one fourth of its length.
In pictures number 4 and 5 the red arrows indicate the whitish front feet.
In picture number 6 the red arrows indicate both morphological traits, short tail with an abnormal curve, and whitish front feet.
